# Fast SARS-CoV-2 Variant Detection Using Snapback Primer High-Resolution Melting

**DOI:** 10.3390/diagnostics11101788

**Published:** 2021-09-28

**Authors:** Joseph C. Lownik, Jared S. Farrar, Grayson W. Way, Angela McKay, Pavitra Roychoudhury, Alexander L. Greninger, Rebecca K. Martin

**Affiliations:** 1 Department of Pathology and Laboratory Medicine, Cedars–Sinai Medical Center, Los Angeles, CA 90048, USA; joseph.lownik@cshs.org; 2 Center for Clinical and Translation Research, Virginia Commonwealth University, Richmond, VA 23298, USA; farrarj@vcu.edu (J.S.F.); waygw@vcu.edu (G.W.W.); 3 Department of Laboratory Medicine and Pathology, University of Washington Medical Center, Seattle, WA 98102, USA; amckay@uw.edu (A.M.); proychou@uw.edu (P.R.); agrening@uw.edu (A.L.G.); 4 Department of Microbiology and Immunology, Virginia Commonwealth University, Richmond, VA 23298, USA

**Keywords:** SARS-CoV-2, high-resolution melting, COVID-19, PCR, COVID testing, snapback primer

## Abstract

SARS-CoV-2, the virus responsible for COVID-19, emerged in late 2019 and has since spread throughout the world, infecting over 200 million people. The fast spread of SARS-CoV-2 showcased the need for rapid and sensitive testing methodologies to help track the disease. Over the past 18 months, numerous SARS-CoV-2 variants have emerged. Many of these variants are suggested to be more transmissible as well as less responsive to neutralization by vaccine-induced antibodies. Viral whole-genome sequencing is the current standard for tracking these variants. However, whole-genome sequencing is costly and the technology and expertise are limited to larger reference laboratories. Here, we present the feasibility of a fast, inexpensive methodology using snapback primer-based high-resolution melting to test for >20 high-consequence SARS-CoV-2 spike mutations. This assay can distinguish between multiple variant lineages and be completed in roughly 2 h for less than $10 per sample.

## 1. Introduction

First discovered in late 2019, the SARS-CoV-2 virus has spread throughout the world and is responsible for the current COVID-19 pandemic. Since it was first sequenced in January 2020, the SARS-CoV-2 virus has diverged into several clades and variants [1]. Over the past year, millions of SARS-CoV-2 sequences have been made publicly available and spurred intense research into the importance of specific mutations, particularly in the gene encoding the Spike protein [1,2,3]. The Spike protein is responsible for viral entry into host cells and is the target of all current approved vaccines.

In late 2020, the B.1.1.7 variant emerged in the United Kingdom and early studies suggested increased transmissibility and mortality associated with this variant [4]. The B.1.1.7 variant contains several mutations in the Spike gene. Additionally, the P.1 and B.1.351 strains which have emerged in Brazil and South Africa, respectively, contain additional mutations in the Spike gene and the N-terminal domain (NTD) supersite which allow for viral evasion from several monoclonal antibody treatments as well as convalescent plasma from both infected and vaccinated patients [5,6,7,8,9]. Mutations at E484 in the Spike gene seen in B.1.351, P.1, and, recently, B.1.617.2 variants are particularly concerning for the lack of protection with vaccination associated with these mutations [10,11,12].

Viral whole-genome sequencing has been the primary mechanism to identify variant spread and novel mutations and lineages. While viral whole-genome sequencing is a comprehensive approach for variant detection, it can be both cost- and time-prohibitive for institutions outside of genome centers and reference laboratories [13]. To address this issue, several groups have attempted to develop PCR- (polymerase chain reaction) based methods for clade or variant determination using either allele-specific probes, small amplicon high-resolution melting, fluorescence resonance energy transfer polymerase chain reaction, or CRISPR [14,15,16,17,18]. However, these studies focused on a limited number of regions in the SARS-CoV-2 genome, allowing for only minimal identification of clades and variants and targeted only a few relevant spike gene mutations. Additionally, a few companies have released variant detection reverse transcription polymerase chain reaction (RT-PCR) assays, albeit at a high cost.

To address these issues, we set out to develop an inexpensive RT-PCR assay to identify variants and classify Spike gene mutations that have been shown to result in structural changes in the Spike protein. These mutations affect either transmissibility or neutralizing antibody effectiveness. In this paper, we present 21 individual assays which are able to identify >20 known mutations as well as potentially identify more novel mutations based on assay coverage. Additionally, these assays can be conducted on a single RT-PCR run utilizing 24 wells and can be completed in ~2 h. Overall, we present a rapid, inexpensive methodology ideal for variant screening and mutational analysis of SARS-CoV-2.

## 2. Materials and Methods

### 2.1. Samples and Sequencing

This study was approved by the University of Washington Institutional Review Board (STUDY00000408). Nasal and nasopharyngeal specimens were collected into 3 mL PBS prior to SARS-CoV-2 qRT-PCR testing on FDA authorized Roche cobas, Hologic Panther Fusion, or Abbott Alinity m platforms [19]. RNA was extracted from positive specimens using the Qiagen BioRobot or Roche MP96 and libraries were prepared for SARS-CoV-2 whole-genome sequencing using the Illumina COVID-Seq or Swift Biosciences v2 SARS-CoV-2 Panel [20]. SARS-CoV-2 consensus genomes were called using https://github.com/greninger-lab/covid_swift_pipeline (accessed on 22 July 2021). Additional purified SARS-CoV-2 genomic RNA was acquired from Biodefense and Emerging Infections Research Resources (BEI Resources) (Manassas, VA, USA). All samples with GISAID IDs can be found in Table 1.

### 2.2. RNA Extraction for RT-PCR Assay

For clinical specimens collected in all liquid media, RNA was extracted using a PureLink RNA Mini Kit (ThermoFisher, Waltham, MA, USA). The manufacturer’s protocol for RNA isolation from whole blood was used. Briefly, 100 µL of transport media was mixed with 100 µL of Lysis Buffer and vortexed for 15 s followed by centrifugation at 12,000× *g* for 2 min. The supernatant was collected and mixed with 1.5 volumes of 100% EtOH and vortexed. This solution was then added to the column and the manufacturer’s standard protocol for washing and eluting was followed. Confirmation of SARS-CoV-2 RNA was tested using the CDC’s N1 primer/probe assay [21].

### 2.3. Genotyping/Mutation Panel Design

For all mutations, multiple sequences of each variant were acquired from the Global initiative on sharing all influenza data (GISAID) database. The Wuhan-1 strain was used as the reference strain for all assay designs. Sequences were aligned using MAFFT [22,23] and visualized using Benchling (www.benchling.com, accessed on 21 May 2021). Mutations to target were selected using a variety of resources based on the ability to discern individual variants based on combinations of mutations examined as well as choosing mutations which are biologically relevant and characterized to be involved in either increased transmissibility, increased mortality, or ability to not be neutralized by convalescent or immunized serum.

### 2.4. Primer Design

For snapback [24,25] primer design, primers were designed such that the T_m_ of the limiting primer was greater than that of the T_m_ of the excess primer by at least 4 °C [26,27]. Snapback and unlabeled probe assay primers were designed using the NCBI PrimerBlast software suite [28] using the Wuhan-1 SARS-CoV-2 reference strain [29]. Snapback duplex melting temperatures were estimated using mFold [30]. Snapback duplexes were designed to have melting temperature ~10–15 °C below the main amplicon melting temperature (estimated using uMelt [31]). Snapback probe melting temperatures were adjusted by varying the hairpin loop length as well as hybridization duplex length to achieve optimal melting profiles when possible. All primers were ordered from Eurofins Genomics (Lexington, KY, USA) using standard desalting purification. All primer sequences can be found in Table 2.

### 2.5. RT-PCR and HRM

Luna Probe One-Step RT-qPCR 4X Mix with UDG (NEB) was used for all reverse transcription and PCR. All RT-PCR was conducted on a QuantStudio 3 (ThermoFisher) 96-well system with 0.2 mL plates. Primers with snapback hybridization probes were used at 1000 nM and non-hybridization probe primers were used at 50 nM. Additional RT primers for E484 and N501Y were used at 50 nM. All reactions were conducted in 10 µL reactions with 3 µL of purified RNA added for each reaction. RT-PCR thermocycling parameters were as follows: 55 °C for 10 min, 60 °C for 5 min, and 95 °C for 2 min followed by 70 cycles of 95 °C for 1 s, 55 °C for 5 s, and 68 °C for 20 s. Melt curve was started with a denaturation at 95 °C for 5 s followed by rapid cooling to 40 °C with a 20 s hold. Melting data acquisition was set to 10 data points per °C.

### 2.6. Data Analysis

Melt curves were analyzed with uAnalyze 2.1 (www.dna-utah.org, accessed on 21 May 2021). Exponential normalization was used with the normalization area set to include both snapback/probe and amplicon areas. Data for derivative plots showing -d(Helicity)/d(Temp) were generated using uAnalyze 2.1 (www.dna-utah.org, accessed on 21 May 2021) and were plotted using GraphPad Prism 9.0 for publication figures.

## 3. Results

### 3.1. Primer Design

Throughout the emergence of new SARS-CoV-2 lineages, several mutations have developed, particularly in the spike protein. While some of these mutations are unique to individual lineages, there are several mutations which have independently developed in different lineages, suggesting a selective pressure driving these mutations. With this in mind, we set out to develop a panel of assays to target lineage-defining SARS-CoV-2 spike gene mutations well as other mutations which have been shown to be important in neutralizing antibody recognition. Our panel targets >20 mutational patterns in the spike gene.

For our assays, we used snapback primer genotyping with HRM due to the excellent sensitivity and specificity for single nucleotide variants (SNV), deletions, and insertions [32]. Additionally, the use of snapback primers allowed for the examination of multiple mutations within up to ~30 bases from each other. Snapback primer genotyping is advantageous over probe-based genotyping due to its lower cost, as primers have no modifications, and generalizability. Snapback primer genotyping can be conducted on almost any qPCR instrumentation. Primers used for these experiments can be found in Table 2.

Snapback primer genotyping required asymmetric PCR to increase the signal of the snapback hybridization probe on melting. A concentration of 20:1 (1000 nM:50 nM) excess to limiting primer ration was chosen. The excess primer contained the snapback hybridization probe [27]. Forward and reverse primers were designed to bind in a region with lower mutational rate as observed by entropy using NextStrain analysis [33].

Targeting regions for snapback probes were chosen to span the targeted mutations with the mutations being at least 6 bp from either end of the snapback hybridization probe. Snapback hybridization probe melting temperatures were estimated using mFold. The length of the hybridization probe and hairpin length was varied in attempts to achieve a melting temperature ~10–15 °C below the full amplicon melting temperature. Melting temperatures of snapback hybridization probes were also optimized to have >3 °C shift between SNVs when possible.

### 3.2. Optimization and Initial Validation

**Figure 1:**
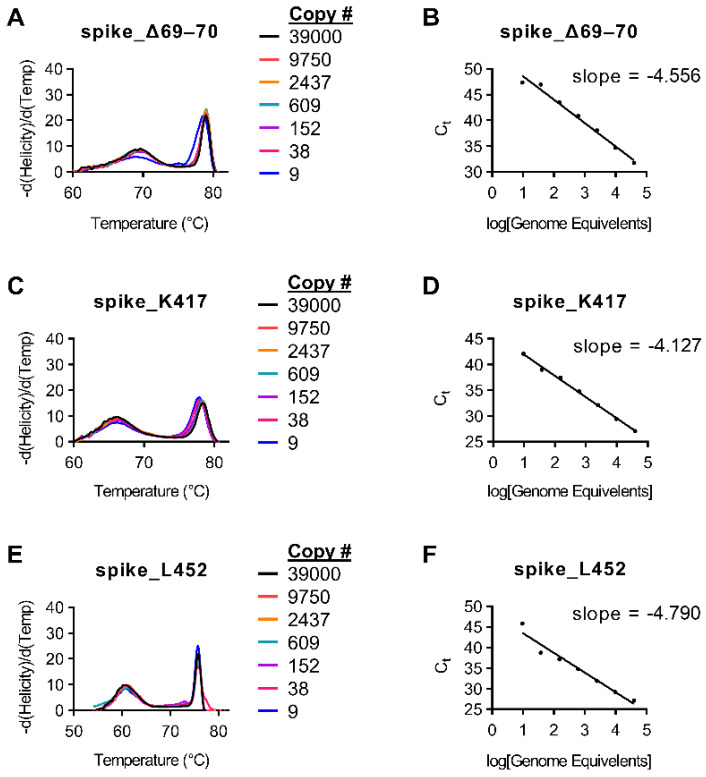
Standard curves for variant snapback HRM assays. Indicated SARS-CoV-2 genomic copy numbers were amplified using the protocol found in Materials and Methods. (**A**) Melt curves for the spike_del69–70 assay with indicated copy number numbers. (**B**) Ct values for the spike_del69–70 assay relative to copy number. (**C**) Melt curves for the spike_K417 assay with indicated copy number numbers. (**D**) Ct values for the spike_K417 assay relative to copy number. (**E**) Melt curves for the spike_L452 assay with indicated copy number numbers. (**F**) Ct values for the spike_L452 assay relative to copy number.

Initial amplification tests for primers were conducted on purified genomic SARS-CoV-2 RNA (BEI; NR-52504). Multiple primer sets were tested for each target. Ct values were acquired using RT-PCR and were compared to initial Ct values using the CDC N1 probe. Sample drop-off was observed for snapback hybridization probes for some targets when the initial sample Ct value of the CDC N1 assay was >32 (<36 copies per reaction; Figure 1 and Supplemental Figure S1).

To conduct asymmetric PCR, several factors needed to be considered. Following exponential amplification in which both primers were utilized equally, linear amplification continues to produce a single-stranded product based on the directionality of the excess primer (which contains the snapback hybridization probe). Because of the linear nature of the amplification of this portion of the reaction, cycle counts are often extended past that of normal PCR. The increase cycle counts can lead to increased non-specific amplification, requiring optimization and evaluation of each individual assay.

**Figure 2:**
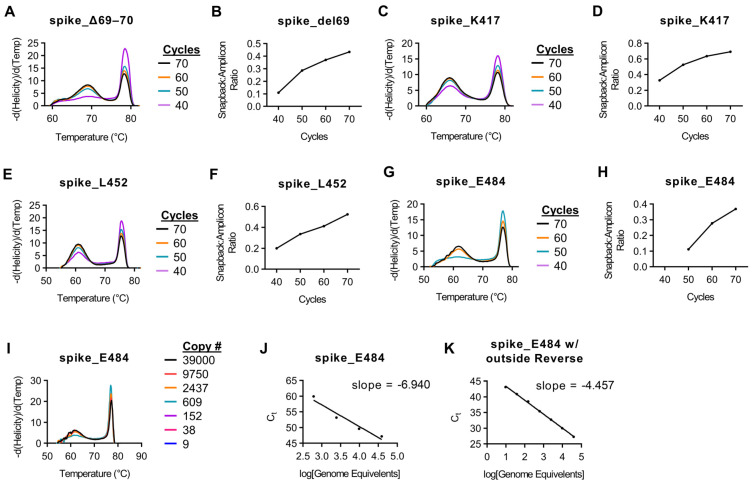
Effect of cycle number on snapback hybridization signal. Indicated SARS-CoV-2 genomic copy numbers were amplified using the protocol found in Materials and Methods with the cycle number varied. (**A**) Melt curves for the spike_del69–70 assay with indicated cycle numbers prior to HRM. (**B**) Snapback primer peak signal to main amplicon peak signal relative to cycle number for the spike_del69–70 assay. (**C**) Melt curves for the spike_K417 assay with indicated cycle numbers prior to HRM. (**D**) Snapback primer peak signal to main amplicon peak signal relative to cycle number for the spike_K417 assay. (**E**) Melt curves for the spike_L452 assay with indicated cycle numbers prior to HRM. (**F**) Snapback primer peak signal to main amplicon peak signal relative to cycle number for the spike_L452 assay. (**G**) Melt curves for the spike_E484 assay with indicated cycle numbers prior to HRM. (**H**) Snapback primer peak signal to main amplicon peak signal relative to cycle number for the spike_E484 assay. (**I**) Melt curves for the spike_E484 assay with indicated copy number numbers. (**J**) Ct values for the spike_E484 assay relative to copy number. (**K**) Ct values for the spike_E484 assay with the addition of an outside reverse primer relative to copy number.

To optimize this reaction, adequate fluorescence intensity of the snapback hybridization probe signal is needed. To accommodate this, we examined melt curves of targets following 40, 50, 60, and 70 cycles of asymmetric PCR. For each assay, the peak height (-d(Helicity)/d(Temp)) is relative to total product when the assays are amplified, melted, and analyzed together. With this in mind, we analyzed the peak height of both the main amplicon as well as the peak of the snapback hybridization probe signal. The ratio of hybridization probe signal to main amplicon signal increased with each successive 10 cycles and started to plateau at 70 cycles for most assays (Figure 2, Supplemental Figure S2).

Using our initial protocol with a reverse transcription step for 5 min at 55 °C, we had difficulty amplifying products for both E484 and N501 targets successfully. Upon further analysis, we observed that the region 150 bp in both 5′ and 3′ directions from these targets had significant secondary structure at 55 °C (Supplemental Figure S3). This secondary structure was slightly decreased in the reverse primer binding site at 60 °C (Supplemental Figure S4). For this reason, we chose to add an additional 5 min to our reverse transcription step at 60 °C as well as add an additional reverse primer ~150–200 bp upstream from the target sites in an area with reduced secondary structure at RT temperatures (Supplemental Figures S3 and S4). This extra reverse primer was added at a limiting concentration. Prior to the addition of an extra reverse primer, no amplicon was detected with <60 cycles for the E484 assay (data not shown). However, following the addition of the extra reverse primer, both the main amplicon and snapback hybridization probe were detectable at ≥50 cycles (Figure 2G,H). Additionally, prior to the additional time at 60 °C as well adding the outside reverse primer, the limit of detection for the E484K assay was ~600 copies and was overall very inefficient (Figure 2I,J). However, the addition of these parameters improved the limit of detection to 36 copies per reaction (Figure 2K). The N501 assay had a similar result, albeit not as severe as the E484 assay (data not shown).

### 3.3. Variant Detection Using Snapback HRM

**Figure 3:**
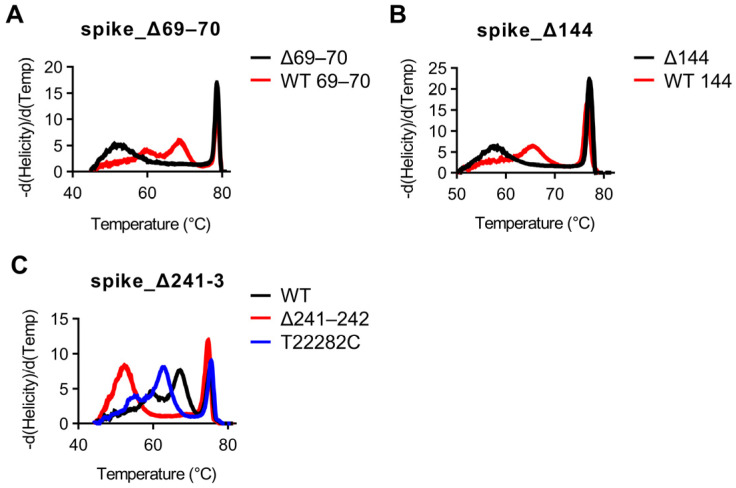
Snapback HRM clearly distinguishes SARS-CoV-2 deletional mutations. (**A**) High-resolution melting plots for the spike_del69–70 assay with WT aa69–70 in red and del69–70 in black. (**B**) High-resolution melting plots for the spike_del144 assay with WT aa144 in red and del144 in black. (**C**) High-resolution melting plots for the spike_del241–3 assay with WT aa241-3 in black, del241–3 in red, and the T22282C SNV in blue.

Using our optimized thermocycling parameters with 70 cycles, we next used SARS-CoV-2 samples which had previously been whole-genome sequenced to test whether snapback primer based HRM would be able to distinguish wild-type vs. mutant sequences. All assays were tested using the Wuhan WT strain as well as all 16 samples. Strains used for validation along with corresponding GISAID accession numbers for positive assays as well as concordances with sequencing results can be found in Table 3. First, mutations involving deletions (del69/70, del144, and del241-243) were examined. All strains with del69/70 mutations were easily discernable by HRM examining the snapback hybridization peak, with concordances with sequencing results of 5/5 for del69/70 and 12/12 for WT aa69/70 (Figure 3A). All strains with del144 were easily discernable by HRM examining the snapback hybridization peak, with concordances with sequencing results of 5/5 for del144 and 12/12 for WT aa144 (Figure 3B). All strains with del241–3 were easily discernable by HRM examining the snapback hybridization peak, with concordances with sequencing results of 3/3 for del241–243 and 14/14 for wild-type aa241–243 (Figure 3B). Snapback hybridization probe melting temperature differences between wild-type and mutant alleles were 16 °C, 8 °C, and 15 °C for the del69/70, del144, and del241–243 assays, respectively (Figure 3A–C). Interestingly, a non-targeted mutation in the snapback hybridization probe region (T22282C) was identified in one of the samples (EPI_ISL_1405252) and was easily distinguishable between wild-type/del241–243 melting profiles (Figure 3C).

**Figure 4:**
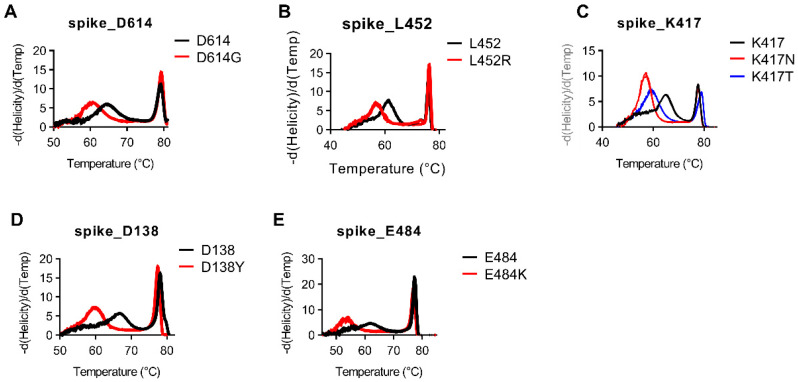
Snapback HRM clearly distinguishes SARS-CoV-2 SNVs. (**A**) High-resolution melting plots for the spike_D614 assay with D614 in black and D614G in red. (**B**) High-resolution melting plots for the spike_L452 assay with L452 black and L452R in red. (**C**) High-resolution melting plots for the spike_K417 assay with K417 in black, K417N in red, and K417T in blue. (**D**) High-resolution melting plots for the spike_D138 assay with D138 in black and D138Y in red. (**E**) High-resolution melting plots for the spike_E484 assay with E484 black and E484K in red.

Besides deletions, the majority of the remaining targets are Class 1–3 SNVs. For assays targeting Class 1–3 SNVs, snapback hybridization probe melting temperatures ranged from 2–10 °C difference between normal and mutant alleles (Figure 4, Supplemental Figure S5). As was seen in the del241–243 assay, one of the major benefits of using snapback-based HRM is the ability to detect several different variant sequences using the same assay. We again see this benefit in the assay targeting the Spike protein mutation at K417 in which we are able to easily discern between K417 (11/11; 100%), K417T (3/3; 100%), and K417N (3/3; 100%) in the same assay (Figure 4C). This is highly advantageous compared to most probe-based assays which would require three different assays to detect these mutations. This benefit is also seen in our assay targeting nucleotide mutations involving Spike protein amino acids L18 and T20. Our single assay targeting this region has distinctly different melt profiles for samples with L18/T20 (13/13; 100%), L18F/T20 (1/1; 100%), as well as L18F/T20N (3/3; 100%) (Supplemental Figure S5D).

Classically, Class 4 SNVs are much more difficult to differentiate compared to Class 1–3 SNVs when methodologies such as short amplicon high-resolution melting are used. For the single Class 4 SNV we targeted which codes for the N501Y mutation, we were able to detect a ~2 °C difference in melting temperature between N501 (11/11; 100%) and N501Y (6/6; 100%) variants (Supplemental Figure S5G).

## 4. Discussion

Since the beginning of the COVID-19 pandemic, mutations within the SARS-CoV-2 genome quickly developed. In addition to the D614G mutation noted early on during the pandemic, several other mutations in the Spike gene have resulted in variants which have been characterized to have increased transmissibility and mortality associated with them [5,34]. The increased transmissibility of these variants was demonstrated with the B.1.1.7 variant which, between January and April of 2021, went from an almost non-exist variant in the United States to the dominant strain [4,34].

The current mainstay for genomic surveillance of SARS-CoV-2 is whole and/or targeted viral genome sequencing. While sequencing is able to give in-depth understanding of the virus at a genomic level and monitor for the development of new mutations and/or variants, it is both time-consuming and expensive. For some locations, a large portion of positive SARS-CoV-2 cases is being sequenced, while this rate is very low in other locations. With such a low number of positive cases being sequenced as well as the time requirement of the sequencing pipeline, there is a possibility of new mutations and/or variant pop-up and spread before sequencing catches them, especially in areas with lower sequencing capacity. Also, clustered outbreaks, particularly in vaccinated individuals, require rapid identification of the variants responsible.

While our assay is significantly faster than viral whole-genome sequencing, our assay requires ~24 wells on a PCR plate, which would limit the number of samples to 4 per 96-well plate and 16 for a 384-well plate. This assay requires 70 cycles of PCR, which, depending on the ramping speed of the thermocycler, could vary the time of the assay significantly. Additionally, this study validated snapback primer HRM assays on the Wuhan strain, B.1.1.7, B.1.429, B.1.351, and P.1 strains, and not the B.1.617.2 delta strain which is now the major viral lineage circulating today. However, several of our assays target conserved mutations seen in the B.1.617.2 lineage. We validated each assay on a limited number of samples; B.1.1.7 (5), B.1.351 (3), B.1.427 (5), and P.1 (3) with a range of each target being present in between 1–16 of the samples. A larger validation set, with the inclusion of the B.1.617.2 lineage, would be beneficial.

For these reasons, we developed a broad HRM-based assay for the identification of known Spike gene mutations as well as variant identification. The methodology presented here using snapback primer-based HRM represents a targeted, inexpensive, rapid, and accurate platform for SARS-CoV-2 variant identification. As the primers used for this methodology are unmodified, they are relatively inexpensive and can be rapidly synthesized and tested for new emerging mutations, such as newer mutations being seen in the B.1.617.2 variant, some of which our panel does not cover. On our instrumentation, this assay can be conducted in ~2 h and costs less than 10 dollars per sample for targeted detection of >20 known mutational sites. Different instrumentation may allow for even faster assay time, depending on instrument ramp rates and further optimization. Overall, this study represents a framework for the rapid and inexpensive development of HRM-based assays for SARS-CoV-2 variant detection.

## Figures and Tables

**Table 1:** SARS-CoV-2 Genomes used for Testing and Validation.

ID	Variant	Gisaid_Full_Name	Gisaid_Accession
1	B.1.429	hCoV-19/USA/WA-UW-59058/2021	EPI_ISL_1526972
2	B.1.429	hCoV-19/USA/WA-UW-59065/2021	EPI_ISL_1526976
3	B.1.429	hCoV-19/USA/WA-UW-59066/2021	EPI_ISL_1526977
4	B.1.429	hCoV-19/USA/WA-UW-59071/2021	EPI_ISL_1526980
5	B.1.429	hCoV-19/USA/WA-UW-59075/2021	EPI_ISL_1167208
6	B.1.1.7	hCoV-19/USA/WA-UW-66221/2021	EPI_ISL_1405249
7	B.1.1.7	hCoV-19/USA/WA-UW-66222/2021	EPI_ISL_1405250
8	B.1.1.7	hCoV-19/USA/WA-UW-66224/2021	EPI_ISL_1405252
9	B.1.1.7	hCoV-19/USA/WA-UW-66227/2021	EPI_ISL_1405254
10	B.1.1.7	hCoV-19/USA/WA-UW-66233/2021	EPI_ISL_1405258
11	B.1.351	hCoV-19/USA/WA-UW-68007/2021	EPI_ISL_1498038
12	B.1.351	hCoV-19/USA/WA-UW-68536/2021	EPI_ISL_1523813
13	B.1.351	hCoV-19/USA/WA-UW-69652/2021	EPI_ISL_1524011
14	P.1	hCoV-19/USA/WA-UW-67962/2021	EPI_ISL_1497947
15	P.1	hCoV-19/USA/WA-UW-67977/2021	EPI_ISL_1498033
16	P.1	hCoV-19/USA/WA-UW-67978/2021	EPI_ISL_1498029

**Table 2:** Primers used in this study.

Targeted Mutation	AA	Forward (5′→3′)	Reverse (5′→3′)	Reverse_Outside (5′→3′)
L5F	5	gc**GCAATAAAACAAGAAAAACAA**ACAACAGAGTTGTTATTTCT	TGTCAGGGTAATAAACACCACG	none
L18F	18	gg**GAGTTCTGGTTGTAAGATTAAC**ACAACAGAGTTGTTATTTCT	TGTCAGGGTAATAAACACCACG	none
T20N	20			
P26S	26	gg**AGTGTATGCAGGGGGTA**AACAACAGAGTTGTTATTTCT	TGTCAGGGTAATAAACACCACG	none
Δ69/70	69	ga**CCAGAGACATGTATAGCATGGAA**GGACTTGTTCTTACCTTTCT	GTTAGACTTCTCAGTGGAAGCA	none
D80	80	cc**ACAGGGTTATCAAACCTCTT**GGACTTGTTCTTACCTTTCT	GTTAGACTTCTCAGTGGAAGCA	none
D138Y	138	ca**ACCCAAAAATGGATCATTACAA**TGTTGTTATTAAAGTCTGTGA	ACCCTGTTTTCCTTCAAGGTCC	none
Δ144	144	cg**TGTTTTTGTGGTAATAAACACCC**TGTTGTTATTAAAGTCTGTGA	AGGTCCATAAGAAAAGGCTGA	none
Δ241/242/243	241	gg**AACTTCTATGTAAAGCAAGTAAAGTTTGA**TGCCAATAGGTATTAACATCAC	ACCTGAAGAAGAATCACCAGGAGTC	none
K417T	417	gg**CAGCAATCTTTCCAGT**TTAGAGGTGATGAAGTCAGA	TCCAAGCTATAACGCAGCCT	none
L452R	452	AGGCTGCGTTATAGCTTGGA	cc**TAATTATAATTACCTGTATAGATTG**TCAGTTGAAATATCTCTCTC	none
E484K	484	gg**AAAACCTTCAACACCATTA**TCAAACCTTTTGAGAGAGAT	TGGAAACCATATGATTGTAAAGGAA	GTTCTTACTGAGTCTAACAAAAA
N501Y	501	aa**AACACCATAAGTGGGT**GTAGCACACCTTGTAATGG	ACAGTTGCTGGTGCATGTAG	GTTCTTACTGAGTCTAACAAAAA
D614G	614	cg**GCAGTTAACATCCTGA**ACACCAGGAACAAATACTTC	TGCATGAATAGCAACAGGGACT	none
Q677H	677	gg**AGTCTGAGTCTGATAAC**GTTTAATAGGGGCTGAACAT	ACCAAGTGACATAGTGTAGGCA	none
P681H	681	gc**CCCGCCGATGAGAATTA**GTTTAATAGGGGCTGAACAT	ACCAAGTGACATAGTGTAGGCA	none
V1176F	1176	gg**TTTGAATGTTTACAACTGAAGCATTAATGC**CTCATTCAAGGAGGAGTTAG	TCATTGAGGCGGTCAATTTCT	none

Bold font denotes snapback hybridization region. Arrow denotes directionality.

**Table 3:** Assay Validation Results.

Mutation	Positive Concordance	Negative (WT) Concordance	Positive Samples GISAID Accessions
L5F	1/1 (100%)	16/16 (100%)	EPI_ISL_1405252
L18F	1/1 (100%)	16/16 (100%)	EPI_ISL_1405258
L18F + T20N	3/3 (100%)	13/13 (100%)	EPI_ISL_1497947, EPI_ISL_1498033, EPI_ISL_1498029
P26S	3/3 (100%)	14/14 (100%)	EPI_ISL_1497947, EPI_ISL_1498033, EPI_ISL_1498029
Δ69-70	5/5 (100%)	12/12 (100%)	EPI_ISL_1405249, EPI_ISL_1405250, EPI_ISL_1405252, EPI_ISL_1405254, EPI_ISL_1405258
D80	3/3 (100%)	14/14 (100%)	EPI_ISL_1405258, EPI_ISL_1498038, EPI_ISL_1523813
D138Y	3/3 (100%)	14/14 (100%)	EPI_ISL_1497947, EPI_ISL_1498033, EPI_ISL_1498029
Δ144	5/5 (100%)	12/12 (100%)	EPI_ISL_1405249, EPI_ISL_1405250, EPI_ISL_1405252, EPI_ISL_1405254, EPI_ISL_1405258
Δ241-3	3/3 (100%)	14/14 (100%)	EPI_ISL_1498038, EPI_ISL_1524011, EPI_ISL_1523813
K417T	3/3 (100%)	11/11 (100%)	EPI_ISL_1497947, EPI_ISL_1498033, EPI_ISL_1498029
K417N	3/3 (100%)	11/11 (100%)	EPI_ISL_1498038, EPI_ISL_1524011, EPI_ISL_1523813
L452R	5/5 (100%)	12/12 (100%)	EPI_ISL_1526972, EPI_ISL_1526976, EPI_ISL_1526977, EPI_ISL_1526980, EPI_ISL_1167208
E484K	4/4 (100%)	13/13 (100%)	EPI_ISL_1405258, EPI_ISL_1497947, EPI_ISL_1498033, EPI_ISL_1498029
N501Y	11/11 (100%)	6/6 (100%)	EPI_ISL_1405249, EPI_ISL_1405250, EPI_ISL_1405252, EPI_ISL_1405254, EPI_ISL_1405258, EPI_ISL_1498038, EPI_ISL_1524011, EPI_ISL_1523813, EPI_ISL_1497947, EPI_ISL_1498033, EPI_ISL_1498029
D614G	16/16 (100%)	1/1 (100%)	EPI_ISL_1526972, EPI_ISL_1526976, EPI_ISL_1526977, EPI_ISL_1526980, EPI_ISL_1167208, EPI_ISL_1405249, EPI_ISL_1405250, EPI_ISL_1405252, EPI_ISL_1405254, EPI_ISL_1405258, EPI_ISL_1498038, EPI_ISL_1524011, EPI_ISL_1523813, EPI_ISL_1497947, EPI_ISL_1498033, EPI_ISL_1498029
Q677H	0/0 (100%)	17/17 (100%)	-
P681H	5/5 (100%)	12/12 (100%)	EPI_ISL_1405249, EPI_ISL_1405250, EPI_ISL_1405252, EPI_ISL_1405254, EPI_ISL_1405258
V1176F	3/3 (100%)	14/14 (100%)	EPI_ISL_1497947, EPI_ISL_1498033, EPI_ISL_1498029

## Data Availability

All data are available upon request. Sequences for SARS-CoV-2 samples for this study can be found at www.gisaid.org (accessed on 21 May 2021).

## Supplementary Materials

The following are available online at https://www.mdpi.com/article/10.3390/diagnostics11101788/s1, Figure S1: Standard curves for variant snapback HRM assays, Figure S2: Effect of cycle number on snapback hybridization signal, Figure S3: RNA secondary structure flanking spike_E484 and spike_N501 assay at 55 °C, Figure S4: RNA secondary structure flanking spike_E484 and spike_N501 assay at 60 °C, Figure S5: Snapback HRM clearly distinguishes SARS-CoV-2 SNVs.
