# Supplementary material for: Fast SARS-CoV-2 Variant Detection Using Snapback Primer High-Resolution Melting
Source: Diagnostics (Basel). 2021 Sep 28;11(10):1788. doi: 10.3390/diagnostics11101788 (PMC8534650; doi:10.3390/diagnostics11101788)
Supplement: Supplementary file 1 [file diagnostics-11-01788-s001.zip › diagnostics-1366556- Supplemental Figures.pdf]

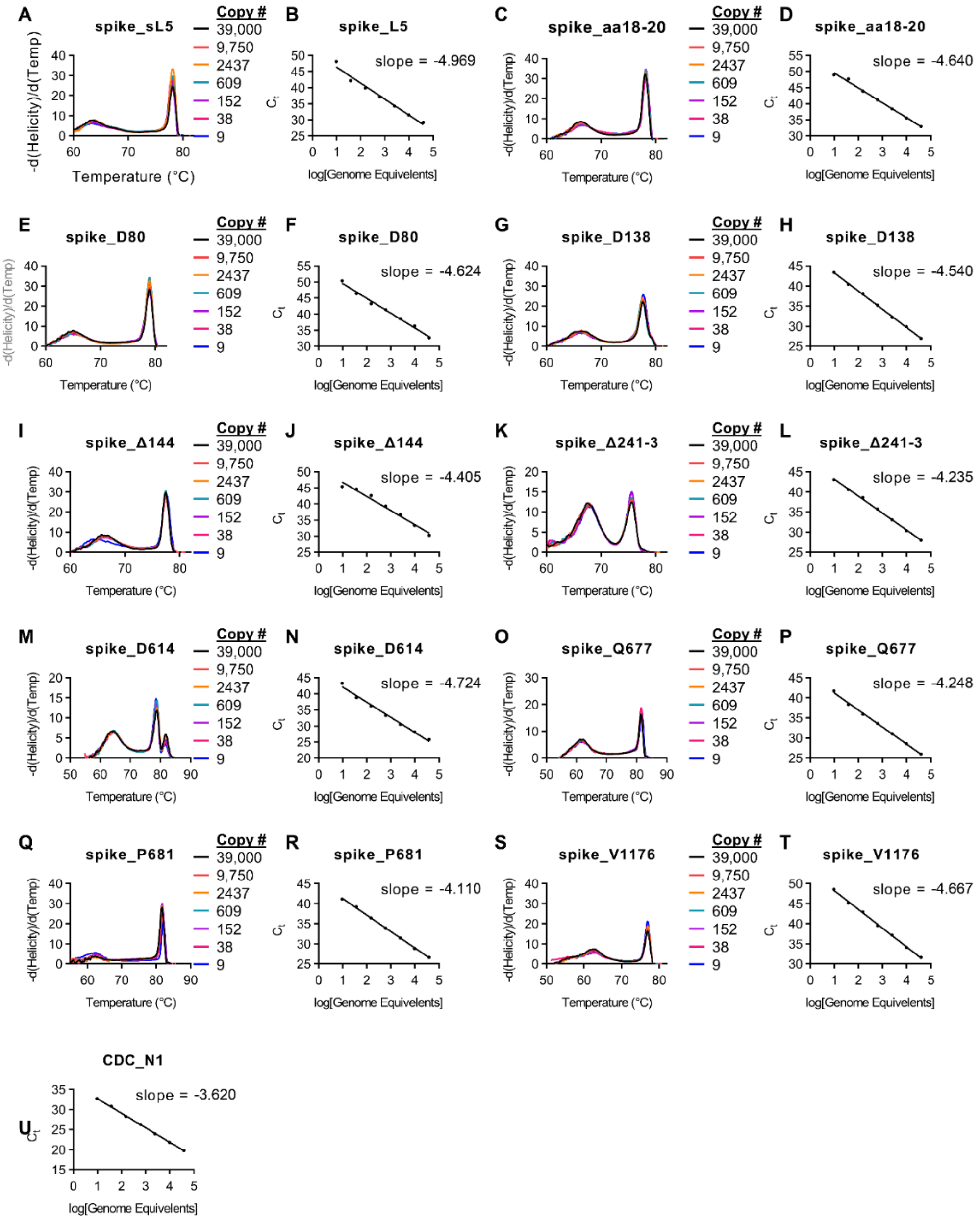

Figure S1. Standard curves for variant snapback HRM assays. Indicated SARS-CoV-2 genomic copy numbers were amplified using the protocol found in Materials and Methods. (A) Melt curves for the spike_L5 assay with indicated copy number numbers. (B) Ct values for the spike_L5 assay relative to copy number. (C) Melt curves for the spike_aa18-20 assay with indicated copy number numbers. (D) Ct values for the spike_aa18-20 assay relative to copy number. (E) Melt curves for the spike_D80 assay with indicated copy number numbers. (F) Ct values for the spike_D80 assay relative to copy number. (G) Melt curves for the spike_D138 assay with indicated copy number numbers. (H) Ct values for the spike_D138 assay relative to copy number. (I) Melt curves for the spike_del144 assay with indicated copy number numbers. (J) Ct values for the spike_del144 assay relative to copy number. (K) Melt curves for the spike_del241-3 assay with indicated copy number numbers. (L) Ct values for the spike_del241-3 assay relative to copy number. (M) Melt curves for the spike_D614 assay with indicated copy number numbers. (N) Ct values for the spike_D614 assay relative to copy number. (O) Melt curves for the spike_Q677 assay with indicated copy number numbers. (P) Ct values for the spike_Q677 assay relative to copy number. (Q) Melt curves for the spike_P681 assay with indicated copy number numbers. (R) Ct values for the spike_P681 assay relative to copy number. (S) Melt curves for the spike_v1176 assay with indicated copy number numbers. (T) Ct values for the spike_V1176 assay relative to copy number. (U) Ct values for the CDC N1 assay relative to copy number.

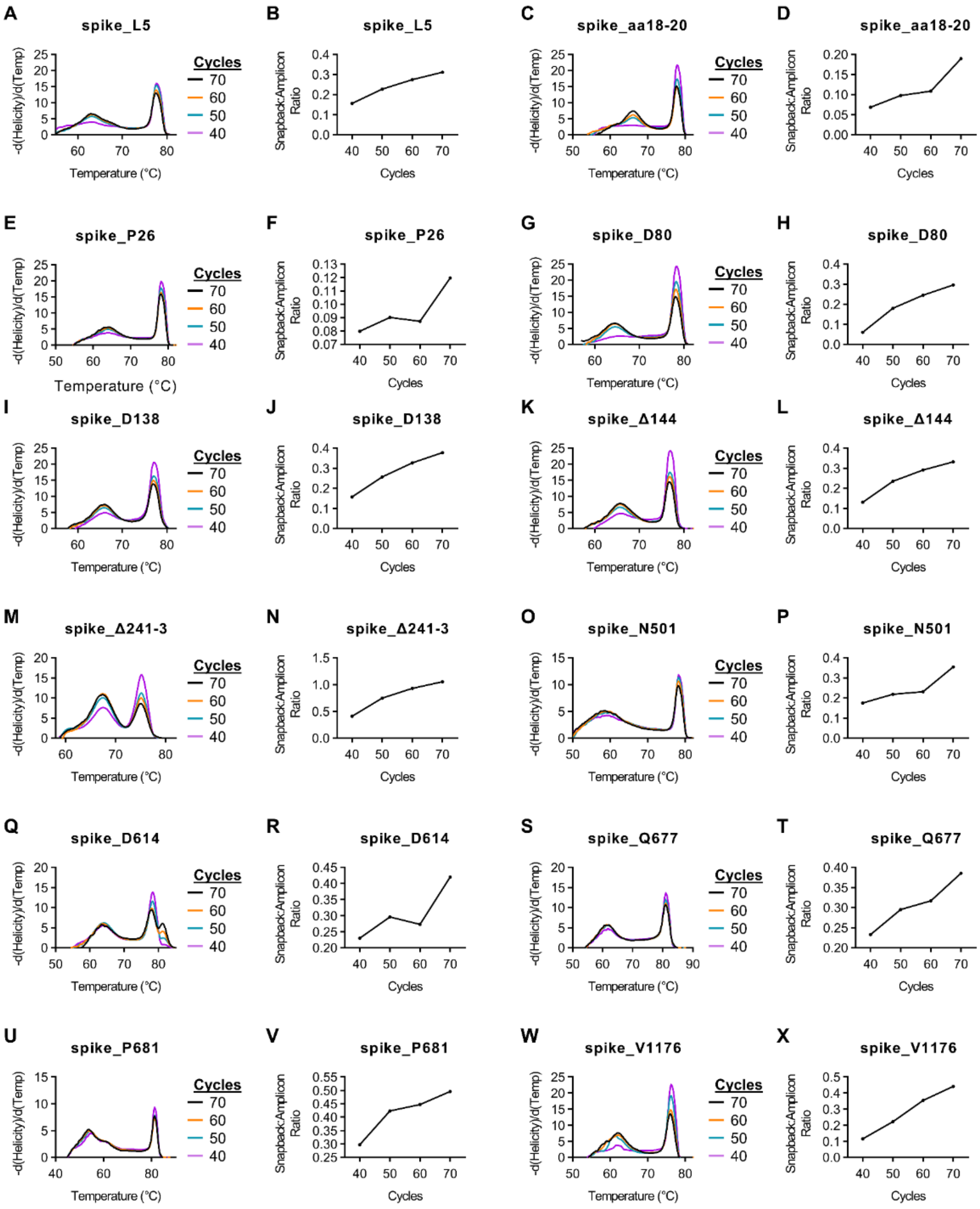

Figure S2. Effect of cycle number on snapback hybridization signal. Indicated SARS-CoV-2 genomic copy numbers were amplified using the protocol found in Materials and Methods with the cycle number varied. (A) Melt curves for the spike_L5 assay with indicated cycle numbers prior to HRM. (B) Snapback primer peak signal to main amplicon peak signal relative to cycle number for the spike_L5 assay. (C) Melt curves for the spike_aa18-20 assay with indicated cycle numbers prior to HRM. (D) Snapback primer peak signal to main amplicon peak signal relative to cycle number for the spike_aa18-20 assay. (E) Melt curves for the spike_P26 assay with indicated cycle numbers prior to HRM. (F) Snapback primer peak signal to main amplicon peak signal relative to cycle number for the spike_P26 assay. (G) Melt curves for the spike_D80 assay with indicated cycle numbers prior to HRM. (H) Snapback primer peak signal to main amplicon peak signal relative to cycle number for the spike_D80 assay. (I) Melt curves for the spike_D138 assay with indicated cycle numbers prior to HRM. (J) Snapback primer peak signal to main amplicon peak signal relative to cycle number for the spike_D138 assay. (K) Melt curves for the spike_del144 assay with indicated cycle numbers prior to HRM. (L) Snapback primer peak signal to main amplicon peak signal relative to cycle number for the spike_del144 assay. (M) Melt curves for the spike_del241-3 assay with indicated cycle numbers prior to HRM. (N) Snapback primer peak signal to main amplicon peak signal relative to cycle number for the spike_del241-3 assay. (O) Melt curves for the spike_N501 assay with indicated cycle numbers prior to HRM. (P) Snapback primer peak signal to main amplicon peak signal relative to cycle number for the spike_N501 assay. (Q) Melt curves for the spike_D614 assay with indicated cycle numbers

prior to HRM. (R) Snapback primer peak signal to main amplicon peak signal relative to cycle number for the spike_D614 assay. (S) Melt curves for the spike_Q677 assay with indicated cycle numbers prior to HRM. (T) Snapback primer peak signal to main amplicon peak signal relative to cycle number for the spike_Q677 assay. (U) Melt curves for the spike_P681 assay with indicated cycle numbers prior to HRM. (V) Snapback primer peak signal to main amplicon peak signal relative to cycle number for the spike_P681 assay. (W) Melt curves for the spike_V1176 assay with indicated cycle numbers prior to HRM. (X) Snapback primer peak signal to main amplicon peak signal relative to cycle number for the spike_V1176 assay.

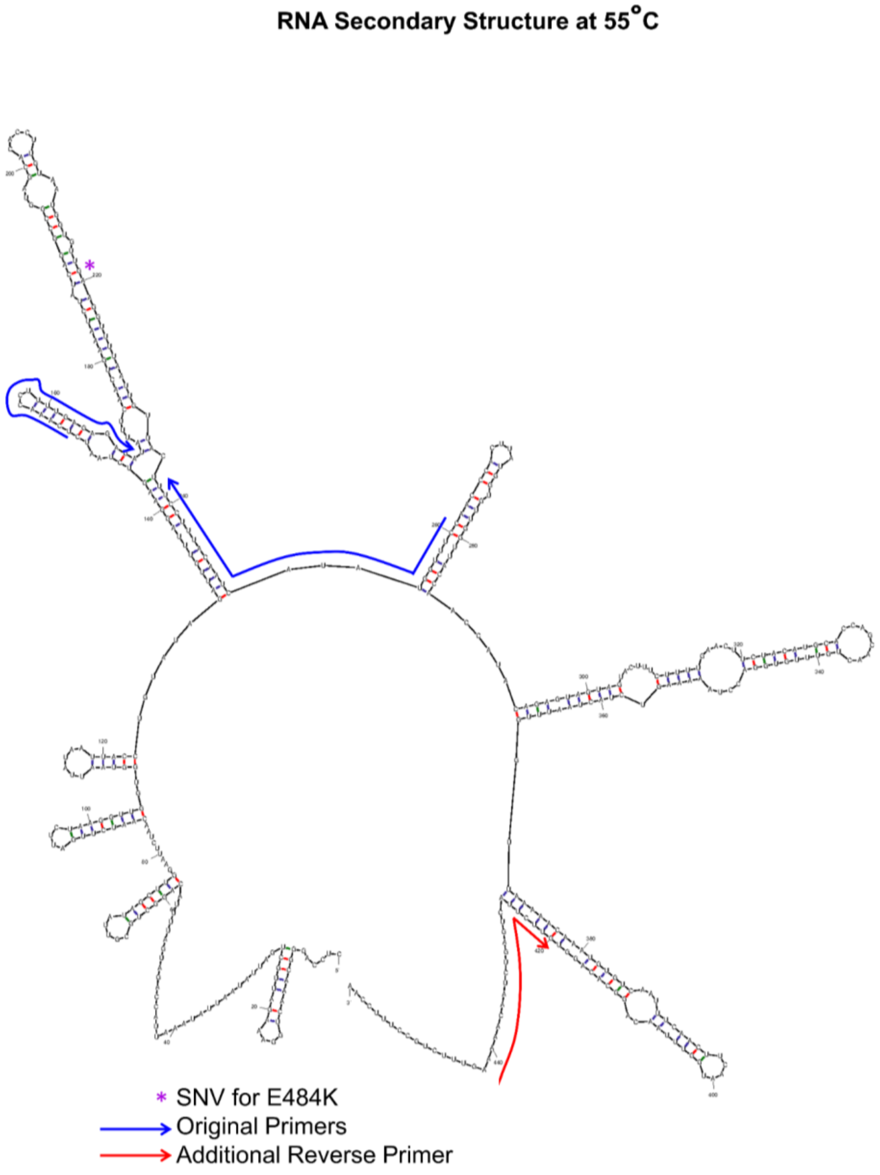

Figure S3. RNA secondary structure flanking spike_E484 and spike_N501 assay at 55 °C. WT SARS-CoV-2 sequence with ~150-200bp on each side of the forward and reverse primers was analyzed for secondary structure using mFold at a temperature of 55°C. Original forward and reverse primers are highlighted in blue. The additional reverse primer added for this assay is highlighted in red. The SNV site for the E484K mutation is marked by a purple asterisk. .

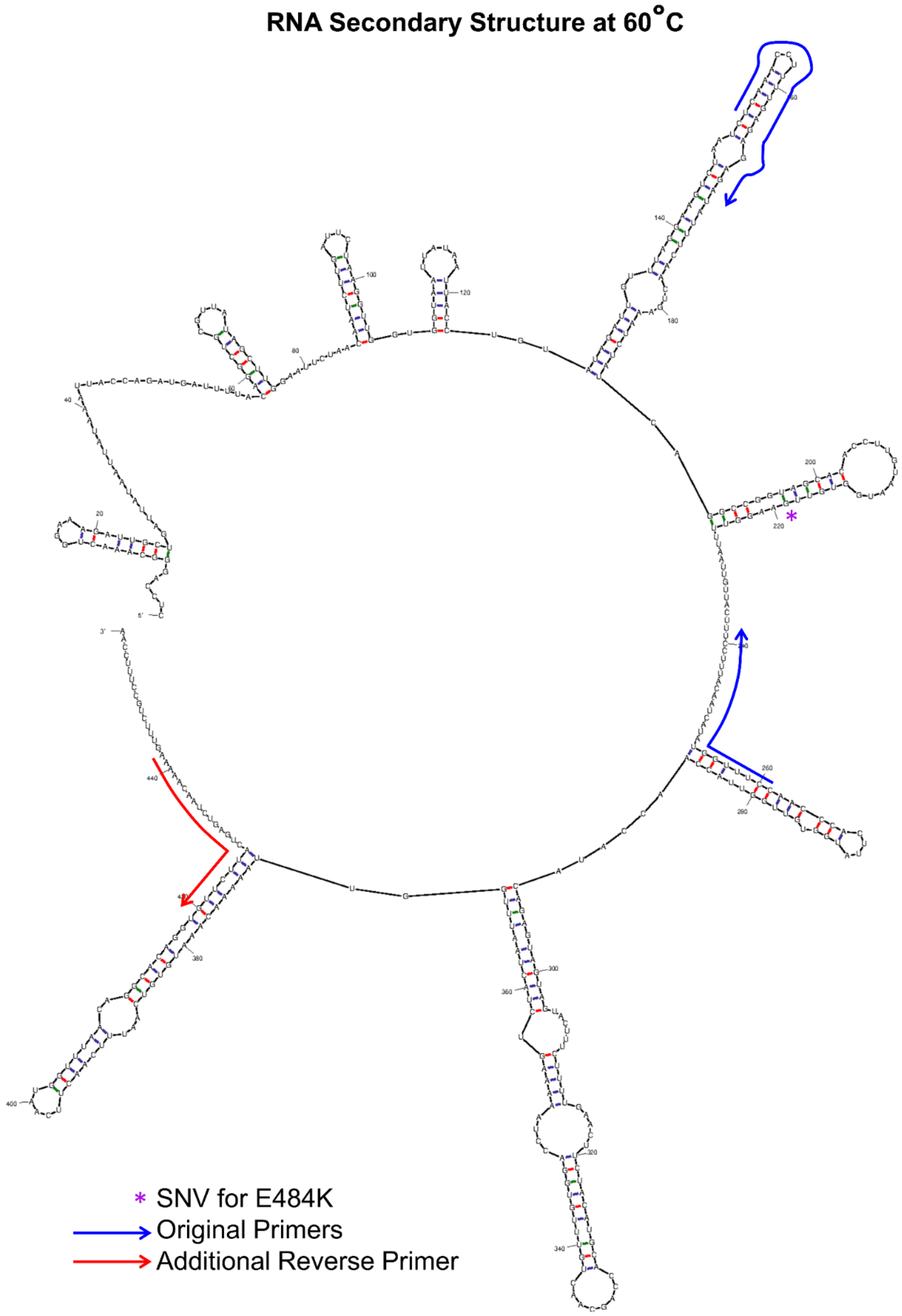

Figure S4. RNA secondary structure flanking spike_E484 and spike_N501 assay at 60 °C. WT SARS-CoV-2 sequence with ~150-200bp on each side of the forward and reverse primers was analyzed for secondary structure using mFold at a temperature of 60°C. Original forward and reverse primers are highlighted in blue. The additional reverse primer added for this assay is highlighted in red. The SNV site for the E484K mutation is marked by a purple asterisk.

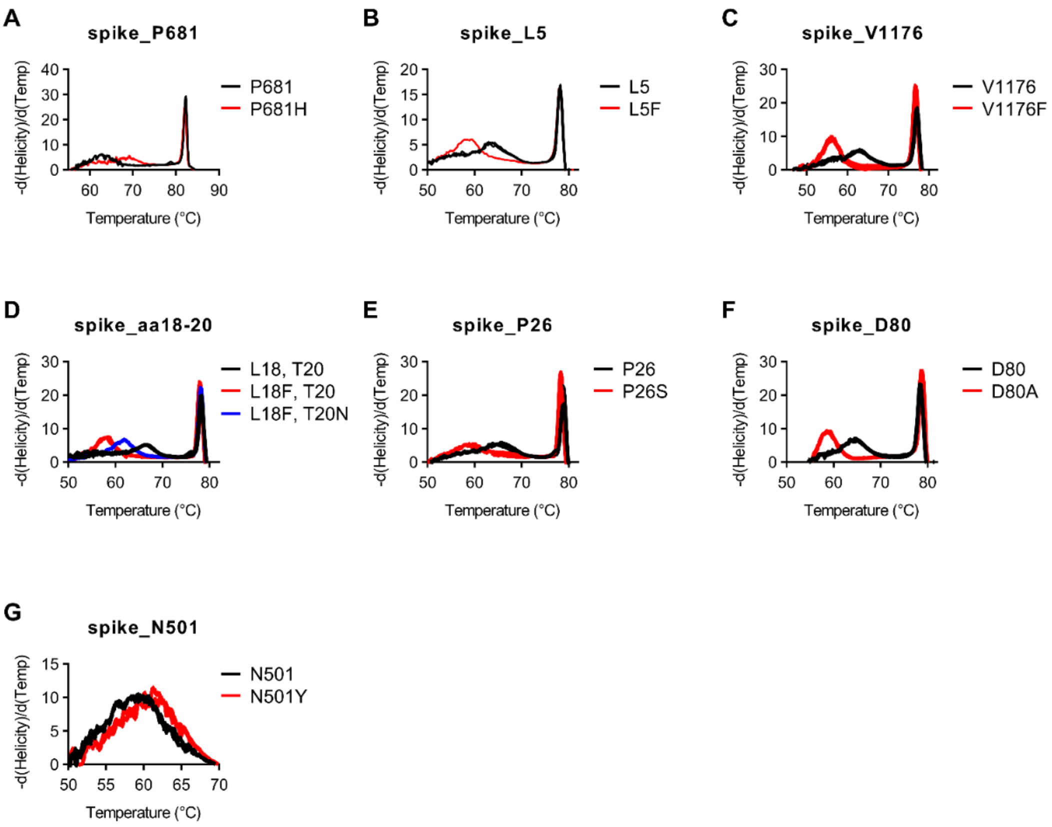

Figure S5. Snapback HRM clearly distinguishes SARS-CoV-2 SNVs. (A) High resolution melting plots for the spike_P681 assay with P681 in black and P681H in red. (B) High resolution melting plots for the spike_L5 assay with L5 black and L5F in red. (C) High resolution melting plots for the spike_V1176 assay with V1176 black and V1176F in red. (D) High resolution melting plots for the spike_aa18-20 assay with WT aa18-20 (L18, T20) in black, L18F T20 in red, and L18F T20N in blue. (E) High resolution melting plots for the spike_P26 assay with P26 in black and P26S in red. (F) High resolution melting plots for the spike_D80 assay with D80 black and D80A in red. (G) High resolution melting plots for the spike_N501 assay with N501 black and N501Y in red, focused on the snapback hybridization melting region.
